# Cervical foraminal stenosis causing unilateral diaphragmatic paralysis without neurologic manifestation: A case report and review of the literature

**DOI:** 10.1097/MD.0000000000021349

**Published:** 2020-09-11

**Authors:** Hyung-Youl Park, Ki-Won Kim, Ji-Hyun Ryu, Chang-Rack Lim, Sung-Bin Han, Jun-Seok Lee

**Affiliations:** a Department of Orthopedic Surgery, Eunpyeong St. Mary's Hospital; b Department of Orthopedic Surgery, Yeouido St. Mary's Hospital, College of Medicine, the Catholic University of Korea, Seoul, Republic of Korea.

**Keywords:** cervical vertebrae, diaphragmatic paralysis, foraminotomy, spondylosis

## Abstract

**Rationale:**

Unilateral diaphragmatic paralysis due to cervical spondylosis has rarely been reported. We present the first case of unilateral diaphragmatic paralysis without radicular pain or motor weakness due to cervical foraminal stenosis and a review of the related literature.

**Patient concerns:**

A 59-year-old man presented with dyspnea and fever. His chest radiograph revealed right hemidiaphragmatic paralysis.

**Diagnoses:**

The differential diagnosis of phrenic nerve palsy excluded mediastinal and neurodegenerative diseases. Imaging studies showed right foraminal stenosis caused by cervical spondylosis at C3–4 and C4–5.

**Interventions:**

The patient underwent foraminotomy at C3–4 and C4–5 on the right side. The operative findings revealed a severe compression of the C4 root.

**Outcomes:**

At 3 months postoperatively, the unilateral diaphragmatic paralysis and dyspnea were recovered.

**Lessons:**

Hemidiaphragmatic paralysis deserves careful evaluation for the presence of cervical spondylosis, even without concomitant neurologic manifestations.

## 1 Introduction

Diaphragmatic paralysis due to cervical spondylosis is extremely rare and several cases have been reported. All reported cases^[1–8]^ presented with respiratory disturbance and neurologic manifestations, including ipsilateral radicular pain or motor weakness.

However, neurologic manifestations may be absent in cases of isolated motor compression of the nerve root that innervates the phrenic nerve.^[4]^ To the best of our knowledge, we present the first case of unilateral diaphragmatic paralysis due to cervical foraminal stenosis without concomitant radicular pain or motor weakness.

## 2 Case report

A 59-year-old man presented with exertional dyspnea that started 1 month earlier. He had undergone wide excision for tongue cancer 6 years ago and no evidence of recurrence was observed.

**Figure 1:**
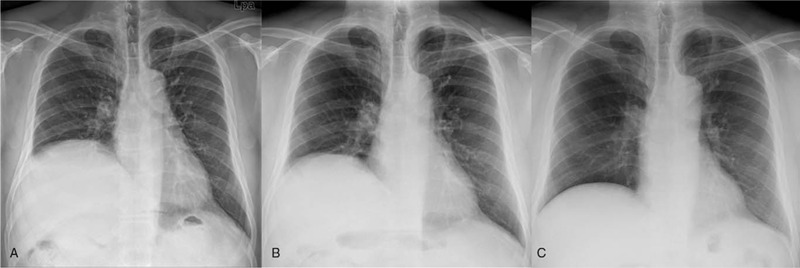
(A) Right hemidiaphragmatic elevation on the initial chest radiograph. (B) No interval change at postoperative 1 month. (C) Remarkable descent of the right diaphragm 3 months after surgery.

A chest radiograph revealed a marked elevation of the right diaphragm compared with the left diaphragm (Fig. 1A). Chest computed tomography (CT) showed no abnormalities of the mediastinal and chest thorax. In pulmonary function tests using a spirometer, his vital capacity was 3.02 L (68% of predicted), forced vital capacity was 3.17 L (71% of predicted), and forced expiratory volume-1 second was 2.17 L (61% of predicted).

The differential diagnosis of unilateral phrenic nerve palsy included thoracic evaluation for lung and mediastinal tumors or infections. Neurologic evaluation for neurodegenerative diseases and imaging studies for recurrent tongue cancer failed to identify the underlying cause. After excluding all possible causes, he was referred for cervical spondylosis evaluation for diaphragmatic paralysis. He did not complain of radiating pain. Motor weakness, sensory changes, and hyperactive deep tendon reflexes were also not observed on neurological examination.

**Figure 2:**
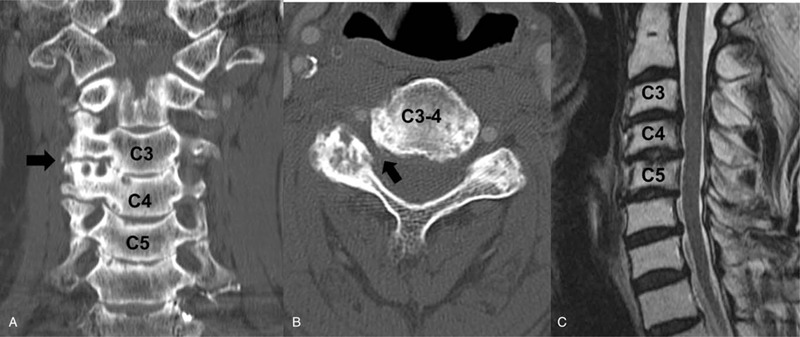
(A) The coronal image and (B) axial image on CT reveal severe foraminal stenosis of C3–4 and C4–5. (C) The sagittal image on MRI showing mild disc protrusion at C3–4–5. CT = computed tomography, MRI = magnetic resonance imaging.

Cervical CT revealed C3–4 and C4–5 foraminal space narrowing due to osteophyte formation (Fig. 2A) and magnetic resonance imaging (MRI) showed that the C4 root was compressed by a bony spur at the right C3–4 foraminal space (Fig. 2B). However, spinal cord compression was not prominent on CT and MRI.

Despite conservative treatment for 1 month, the patient was admitted to the hospital for recurrent fever and dyspnea. Under the diagnosis of hemidiaphragmatic paralysis due to cervical foraminal stenosis, the patient underwent foraminotomies at C3–4 and C4–5 on the right side and the operative findings revealed right C4 root severe compression caused by a bony spur. Foraminal decompression was done until the root passed without resistance.

**Figure 3:**
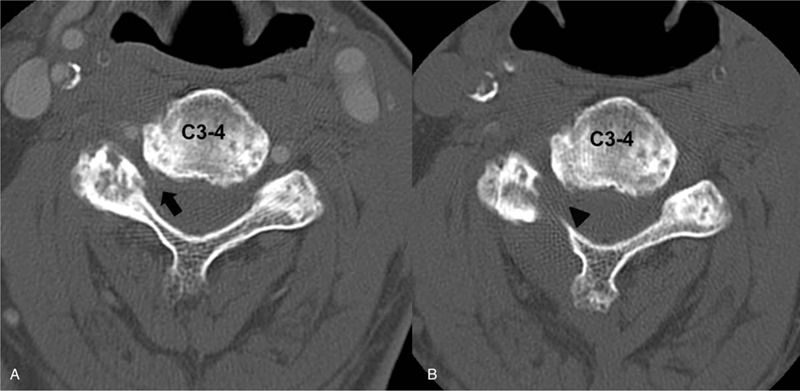
(A) Preoperative foraminal stenosis at C3–4 on the CT axial image. (B) Postoperative foraminal decompression at the C3–4 on the CT axial image. CT = computed tomography.

The chest radiograph demonstrated no interval change at 1 postoperative month (Fig. 1B). At 3 months postoperatively, the chest radiograph revealed a remarkable descent of the right diaphragm compared with the initial radiograph (Fig. 1A and B). The postoperative CT showed a widening of the foraminal canal compared with the preoperative CT (Fig. 3). No dyspnea or pneumonia was observed at the surgical follow-up. The patient has provided informed consent for the publication of this case report and accompanying images.

## 3 Discussion

Diaphragmatic paralysis may develop from various causes and differential diagnosis is important to treat the patients. Upper motor neuron lesions (caused from cerebrovascular accidents, infections, and multiple sclerosis), lower motor neuron lesions (caused from spinal cord injury, poliomyelitis, and amyotrophic lateral sclerosis), phrenic nerve lesions (caused by trauma, surgical damage, tumors, and inflammation), and muscular disorders (such as, myasthenia gravis and muscular dystrophy) should be excluded.^[9,10]^ Thus, thorough physical, neurological, and radiological examinations are the first steps in the differential diagnosis of the causative factors.

Unilateral diaphragmatic paralysis due to cervical spondylosis is rarely reported and to date, only 8 cases have been reported (Table 1).^[1–8]^ All reported cases presented with respiratory disturbances and concomitant neurologic manifestation. However, hemidiaphragmatic paralysis developed in our patient without related neurologic symptoms, such as ipsilateral radicular pain or motor weakness. Weiss et al^[4]^ demonstrated that the motor root may be affected in isolation and patients lack radiating pain or sensory deficits in chronic uncovertebral arthrosis.

**Table 1:**
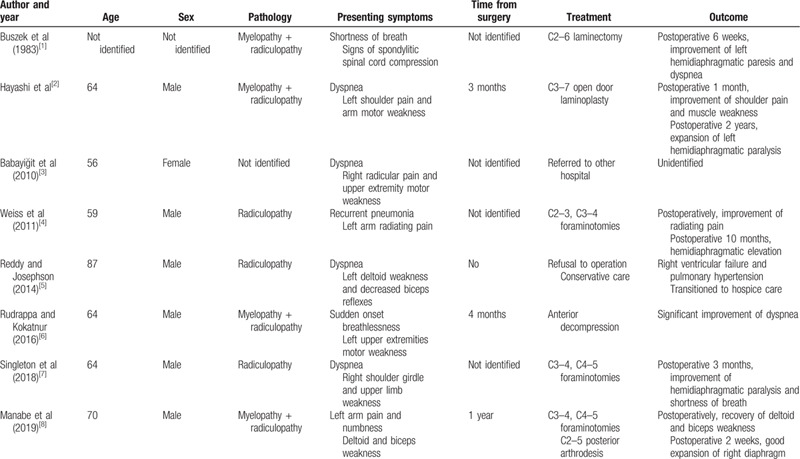
Previous case reports of hemidiaphragmatic paralysis due to cervical spondylosis.

In our case, surgery was cautiously decided because the patient did not complain of related radiating pain or motor weakness. After excluding all possible causes and taking conservative treatment, we finally decided on surgery based on a diagnosis of hemidiaphragmatic paralysis without neurologic manifestations due to cervical foraminal stenosis.^[11]^ To the best of our knowledge, this was the first case of unilateral diaphragmatic paralysis due to cervical foraminal stenosis without concomitant neurologic manifestation.

Recent studies reported that electromyogram (EMG) and nerve conduction studies (NCS) might be helpful to diagnose hemidiaphragmatic paralysis.^[5,8]^ Transcutaneous phrenic nerve stimulation in NCS shows a decrease in amplitude compared with the other side and EMG reveals radiculopathy, suggestive of nerve root compression.^[8]^ Moreover, EMG/NCS might provide evidence of degenerative neuromuscular diseases, such as amyotrophic lateral sclerosis or myopathy.^[5]^ Unfortunately, neither EMG nor NCS were performed in our case.

The phrenic nerve receives its main input from the fourth (C4), and partly the third (C3) and fifth (C5), cervical nerve roots.^[4,12]^ Consequently, diaphragmatic paralysis can be caused by myelopathy at the C2–3 disc level compressing the C4 neuromere or radiculopathy at the C3–4 level compressing the C4 nerve root.^[2,12]^ In the diaphragmatic paralysis, patients with radiculopathy at the C3–4 level, in contrast to patients with myelopathy, might be missed due to ambiguous symptoms and more careful attention should be paid.

Surgery for decompression should be recommended for patients with hemidiaphragmatic paralysis resulting from cervical spondylosis. Reddy et al^[5]^ reported that an 87-year-old male patient with diaphragmatic weakness from cervical spondylosis refused to undergo surgery and transitioned to hospice care 2 years later due to right ventricular failure and pulmonary hypertension, despite conservative care that included biphasic intermittent positive airway pressure and diuretics.

Regarding the surgical approach, posterior foraminotomy is sufficient to decompress the nerve root, as in our patient who presented with radiculopathy due to foraminal stenosis.^[4,7]^ However, open door laminoplasty or laminectomy is considered for cases presenting with myelopathy and radiculopathy^[1,2,13]^ and foraminotomy can be additionally considered for foraminal stenosis remaining after central decompression.^[8]^

In conclusion, cervical spondylosis should be considered in case of unexplained hemidiaphragmatic paralysis after differential diagnosis. Unilateral diaphragmatic paralysis without concomitant radiating pain or muscle weakness may develop due to foraminal stenosis, particularly at C3–4. Our experience demonstrates that satisfactory clinical outcomes may be achieved through a posterior foraminotomy.

## Author contributions

**Conceptualization:** Jun-Seok Lee.

**Data curation:** Hyung-Youl Park, Ji-Hyun Ryu, Chang-Rak Lim, Sung-Bin Han.

**Funding acquisition:** Hyung-Youl Park.

**Investigation:** Hyung-Youl Park, Ki-Won Kim, Ji-Hyun Ryu, Chang-Rak Lim, Sung-Bin Han, Jun-Seok Lee.

**Supervision:** Jun-Seok Lee.

**Writing – original draft:** Hyung-Youl Park, Ki-Won Kim.

**Writing – review & editing:** Ki-Won Kim, Jun-Seok Lee.
